# Variation in physician recommendations, knowledge and perceived roles regarding provision of end-of-life care

**DOI:** 10.1186/s12904-015-0050-y

**Published:** 2015-10-26

**Authors:** Chetna Malhotra, Noreen Chan, Jamie Zhou, Hannah B. Dalager, Eric Finkelstein

**Affiliations:** Lien Centre for Palliative Care, Duke-NUS Graduate Medical School, 8 College Road, Singapore, 169857 Singapore; Program in Health Services and Systems Research, Duke-NUS Graduate Medical School, Singapore, Singapore; Department of Haematology-Oncology, National University Cancer Institute, Singapore, Singapore; Maxwell School of Citizenship and Public Affairs, Syracuse University, Syracuse, NY USA

**Keywords:** Terminal care, Treatment recommendations, Palliative care, Pain management, Perceived roles, Vignettes, Best-worst scaling

## Abstract

**Background:**

There is high variability in end-of-life (EOL) treatments. Some of this could be due to differences in physician treatment recommendations, their knowledge/attitude regarding palliative care, and their perceived roles in treating patients with advanced serious illness (ASI). Thus, the objective of this paper was to identify potential variation in physician recommendations, their knowledge/attitude regarding palliative care and perceived roles in treating ASI patients.

**Methods:**

A cross-sectional survey consisting of vignettes describing patient characteristics that varied by age, expected survival, cognitive status and treatment costs and asked physicians whether they would recommend life-extending treatments for each scenario, was administered to 285 physicians who treat ASI patients in Singapore. Physicians were also assessed on their knowledge/attitude in palliative care. They were administered a best-worst scaling exercise requiring them to select their most and least important role as a physician caring for an ASI patient.

**Results:**

There was a wide variation in physician recommendations for life-extending treatments for patients with similar profiles, which can partly be attributed to physician characteristics (years of experience and place of training). Only about one-fourth of the physicians answered all knowledge/attitude questions correctly. Statements assessing knowledge/attitude regarding pain management had the fewest correct responses. The most important perceived role regarding provision of EOL care concerned symptom management.

**Conclusions:**

Results suggest that variation in physician treatment recommendations may be partly related to their own characteristics, raising concerns regarding the EOL care being provided to patients. Efforts should be made to better understand this variation and to provide the physicians with additional training in key aspects of palliative care management.

**Electronic supplementary material:**

The online version of this article (doi:10.1186/s12904-015-0050-y) contains supplementary material, which is available to authorized users.

## Background

Variation in medical practice is a common phenomenon [1, 2] and although understudied, is likely to be large among patients with advanced serious illnesses (ASI), such as those with advanced cancers or advanced organ failures, for which treatment options are largely restricted to life extension or palliation of symptoms. Such variation can be appropriate and beneficial if it occurs due to differences in illness characteristics or patient preferences. However if the variation in treatment recommendations for these patients, such as whether or not to recommend a life extending treatment, is independent of patient characteristics or preferences, and is due to physician characteristics or their practice style, then this raises questions about whether patients are receiving the best possible care at the end of life (EOL) and whether healthcare resources are being used optimally.

Research documents variation in physician practice styles or recommendations for a range of services and conditions [3–11]. Such variation may relate to differences in cost, [12] patient age, [13] presence of cognitive impairment, [14] health care systems, [3, 4] cultural or health care beliefs, [5] and treatment guidelines [6]. The relationship with patient’s cognitive status may be due to caregiver or physician attitudes regarding perceived benefit of treatment to patients with dementia. Equivalent research for ASI patients in Singapore (the setting for this study), though presently lacking, is likely to indicate that variation in physician practice styles will be large as patients (and caregivers) often defer to their physicians for final decisions regarding treatment [7] and much of the care is paid out-of-pocket [8]. The latter point is especially salient as the lack of a third party payer affords physicians greater flexibility in treatment recommendations, with fewer concerns about coverage, oversight, and reimbursement guidelines.

Variation in treatment of patients with ASI may also result from variation in knowledge/attitude and perceived roles of physicians in managing such patients. In Singapore, most EOL care is provided by non-palliative care physicians who have little training in palliative care [9, 10]. Poor knowledge due to absence of training may result in poor management of patients. Furthermore, control of pain and other symptoms has traditionally received lower priority in care of patients with cancer and other ASI as compared to extending patient’s life [11]. This may be related to (non-palliative care) physicians’ perception of their role in primarily providing life-extending treatments as opposed to managing their symptoms. If so, these physicians may be less likely to provide palliative care to patients.

It is challenging to identify the causes of variation in treatment patterns for patients with ASI in the real world given differences in health status, patient and caregiver preferences, socio-demographics and other factors. Therefore, we attempted to address this issue via a carefully designed survey of physicians who treat patients with ASI in Singapore. The survey aimed to ascertain the variation in physician recommendations for life-extending treatments for ASI patients, their knowledge/attitude regarding palliative care provision and their perceived roles regarding provision of EOL care. We hypothesized that there will be a large variation in physician recommendations, which will systematically vary by their characteristics (age, gender, experience, country of medical training and training in palliative care), that they will lack appropriate knowledge/attitude about palliative care provision and that extending patient’s life for as long as possible will be the most important perceived role of physicians. If confirmed, then this might raise concerns that patients are receiving sub-optimal care at the EOL.

## Methods

### Setting and sample

Between June and December 2013, the study team invited physicians to take the study at departmental meetings for select departments at the four largest public hospitals in Singapore, and sent email requests to physicians via electronic mailing lists maintained by select medical groups. Departments that are expected to receive large numbers of ASI patients, e.g., Medicine, Surgery, Oncology, Cardiology etc. were selected for the study. A convenience sample of 285 (179 on-line and 106 on paper) physicians who treat patients with ASI in Singapore responded to the survey, with the overwhelming majority (93.3 %) coming from public sector hospitals and clinics. Informed written consent was obtained from all participants. The study was approved by the Institutional Review Board at the National University of Singapore. Dataset for this study is available on request. A brief description of Singapore’s health care system is in Additional file 1.

### Survey questionnaire

In the first section of the questionnaire, nine vignettes described a hypothetical patient with ASI. All physicians answered all of the nine vignettes in the survey questionnaire. Previous studies have shown vignettes to be a valid tool in measuring clinical practice variation [12, 13]. Each vignette in our study was identical except for 4 attributes that systematically varied with 3 levels each: patient age (35, 55, 75 years), median life-extension associated with the treatment option (4, 12, 24 months), 5-year survival rate associated with the treatment option (1, 5, 10 %), and cost of the treatment (S$10,000, S$55,000, S$100,000). The specific attribute levels were selected to elicit large variation in responses and were pre-tested before the main survey. The nine vignettes were chosen such that each attribute level appeared three times and only once with every other level from other attributes. This experimental design, generated by Sawtooth SSI web, [14] allows for estimating unbiased main effects of each level on choice of outcome. An example vignette is shown in Table 1, Panel A.

**Table 1:**
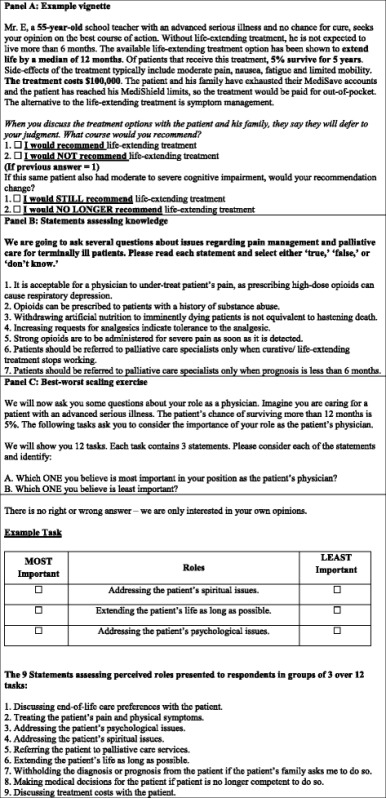
Example vignette, statements assessing knowledge and perceived roles of physicians

In the next section, physicians were presented with seven statements (Table 1, Panel B) to assess their knowledge/attitude regarding palliative care and pain management for patients with ASI and asked to indicate whether or not each statement was true or false. If their response matched with the most appropriate response, it was deemed to be correct. These statements and their most appropriate responses were generated through a review of literature and discussion among investigators and their content was validated by several palliative care physicians.

Finally, physicians were presented with a best-worst scaling exercise to elicit their perception about their most and least important roles in treating a patient at the EOL. Best-worst scaling is a method of eliciting stated preferences that has been found to be superior in differentiating between individual preferences as compared to rating or Likert scales [15–17]. In this method, respondents are asked to make their best and worst choices from a series of choice sets containing 3 or more attributes/items. This method assumes that respondents find it easier to choose between extremes in a set of 3 or more items. In this survey, we identified 9 physician perceived roles from a review of literature and discussion amongst investigators. These were pilot tested prior to fielding. Pilot testing was done to ensure that all statements were clear and relevant for physicians; those unclear or were not chosen as either best or worst in any of the tasks, were subsequently removed or modified. We then used a balanced incomplete block design [17] to create 12 choice sets. A balanced incomplete block design is an experimental design which gives fixed set sizes (in this case the set size was 3), and each attribute/item has equal occurrence and co-occurrence (in this case, each item/role appeared 4 times, every time with two different roles). This helps to minimize any design-induced bias. The roles and an example task are included in Table 1, Panel C.

### Analysis

#### Vignettes

For vignettes, we calculated the percentage of physicians recommending treatment for each of the attribute levels for patients with and without cognitive impairment. We then ran logistic regression model to estimate the effect of each attribute level and physician characteristics (gender, years of experience, palliative care training received and country of basic medical training) on treatment recommendations. Logistic regression was used as the dependent variable (whether or not the physician recommends a life extending treatment) is binary. We tested the model for any specification error (using *linktest* in STATA) and goodness-of-fit (Hosmer and Lemeshow's goodness-of-fit test). Results showed that the model was adequately specified (*linktest* was not statistically significant) and had good fit (p-value for Hosmer and Lemeshow’s test = 0.75). Separate model was run for vignette responses referring to patients with cognitive impairment.

#### Knowledge/attitude questions

For the knowledge/attitude questions, the proportion of physicians who answered correctly on each statement was first calculated. Since the total number of correct responses for each physician is a count variable, a poisson regression [18]. The independent variables in the model included physician gender, years of experience, palliative care training received and country of basic medical training.

#### Best-worst scaling

For the best-worst scaling exercise, we calculated an importance score [19] for each role by subtracting the number of times each role was chosen as being ‘least important’ from the number of times it was chosen as being ‘most important’. As each role appears 4 times in the design, the possible values of these best-worst (B-W) scores at the individual level ranged from −4 to 4. These scores were then rescaled to range between 0 and 1. These importance scores are known to be highly collinear with the estimates from a conventional multinomial logistic regression or conditional logistic regression choice model [19].

## Results

Physician demographics are presented in Table 2. The majority (66 %) received no additional training in palliative care beyond their basic medical school training.

**Table 2:** Physician demographics (*n* = 285)

Characteristics	
*Age (in years)*	
Mean (SD)	33.2 (8.14)
Range	24-–65
	n (%)
*Gender*	
Male	155 (54.4)
Female	129 (45.3)
*Experience*	
7 year or less	164 (57.5)
More than 7 years	121 (42.5)
*Country of Basic Medical Training*	
Singapore	171 (60.0)
Asia (other than Singapore)	53 (18.6)
Europe, N. America, Australia, New Zealand	50 (17.5)
*Additional Training in Palliative Care*	
None	188 (66.0)
Workshop only	53 (18.6)
Certificate, Diploma or Degree	44 (15.4)

### Vignettes

**Fig. 1:**
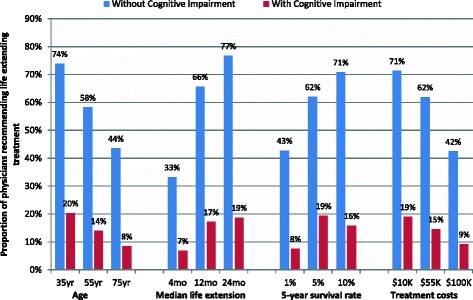
Proportion of physicians recommending life extending treatment for patients with varying characteristics

Figure 1 shows the variation in proportions of physicians recommending life-extending treatment for each attribute level (predicted at the mean of the other levels). We found that treatment recommendations varied widely for any given patient profile unless the patient was assumed to have cognitive impairment. 73 % of physicians recommended life extending treatment for patients in the best-case scenario that consisted of a 35 year old patient, where treatment would extend life by 24 months, 5-year survival rate was 10 % and treatment cost was $10,000. In contrast, 40 % of physicians recommended treatment (and 60 % did not) in the worst case (i.e., oldest patient, highest treatment cost, worst prognosis) scenario that consisted of a 75 year old patient, where treatment would extend life by 4 months, 5-year survival rate was 1 % and treatment cost was $100,000.

We also find some variation in treatment recommendations by age, treatment costs and prognosis even for patients with moderate to severe cognitive impairment, with only 18 % of the physicians recommending treatment in the best-case scenario and 8 % recommending treatment in the worst-case scenario.

Results from logistic regression (Table 3) show that physicians are more likely to recommend life-extending treatments to patients who are younger, have a better prognosis, and if treatment costs are lower. Furthermore, physicians with more years of experience were more likely to recommend life-extending treatment to patients without cognitive impairment, but were less likely to recommend treatments to patients with cognitive impairment. Physicians, trained in Europe, North America, Australia and New Zealand, were more likely to recommend life-extending treatment to patients with cognitive impairment compared to those trained in Singapore.

**Table 3:** Logistic regression model predicting the odds of recommending life extending treatment, for patients without and with cognitive impairment, by patient, treatment and physician characteristics

	Patients without cognitive impairment		Patients with cognitive impairment	
Attribute	Odds ratio	*P*-value	Odds ratio	*P*-value
Age (Reference: 75 years old)				
55 years old	3.217	0.000	2.288	0.001
35 years old	6.130	0.000	4.332	0.000
Median life extension (Reference: 4 months)				
12 months	6.099	0.000	3.321	0.000
24 months	11.810	0.000	3.910	0.000
5-year survival rate (Reference: 1 %)				
5 %	2.165	0.000	1.448	0.130
10 %	3.563	0.000	2.104	0.000
Treatment cost (Reference: $100,000)				
$55,000	2.993	0.000	2.503	0.000
$10,000	5.193	0.000	4.024	0.000
Gender (Reference: male)				
Female	0.956	0.657	0.992	0.947
Years of experience	1.024	0.000	0.986	0.049
Country of Basic Medical Training (Reference: Singapore)				
Asia (other than Singapore)	0.804	0.097	1.034	0.842
Europe / N. America / Australia / New Zealand	1.277	0.067	1.686	0.000
Additional Palliative Care Training (Reference: none)				
Workshops on Palliative Care	0.967	0.799	1.050	0.757
Certificate, Diploma, or Degree in Palliative Care	0.905	0.494	0.944	0.752

### Knowledge/attitude questions

Results of the questions (Table 4) show that the percent of correct answers ranged from 51 to 92 %. The statement assessing prescription of a strong opioid for severe pain as soon as it is detected had the fewest correct responses (51 %). Results from the Poisson regression showed that physicians with greater years of experience (β = 0.02; *p*-value = 0.03), those trained in Europe, North America, Australia, or New Zealand (versus those trained in Singapore; β = 0.67; *p*-value = 0.001) and those with additional training in palliative care (versus those with no additional training; β = 0.47; *p*-value = 0.045) had a greater number of correct responses.

**Table 4:** Proportion of physicians with correct answers to each of the statements assessing their knowledge/ attitude to pain management and aspects of palliative care

True/False Statement	Answer	% Correct
It is acceptable for a physician to under-treat patient’s pain, as prescribing high-dose opioids can cause respiratory depression.	False	92
Opioids can be prescribed to patients with a history of substance abuse	True	87
Withdrawing artificial nutrition to imminently dying patients is not equivalent to hastening death.	True	74
Increasing requests for analgesics indicate tolerance to the analgesic.	False	73
Strong opioids are to be administered for severe pain as soon as it is detected.	True	51
Patients should be referred to palliative care specialists only when curative/ life-extending treatment stops working.	False	89
Patients should be referred to palliative care specialists only when prognosis is less than 6 months.	False	87

### Best-worst scaling

**Fig. 2:**
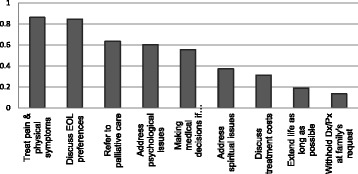
Importance scores on the best-worst scaling exercise

The importance scores from the best-worst exercise showed that contrary to our hypothesis, the most important perceived role in treating a patient at the EOL was treating pain and other physical symptoms, which was rated most important 84 % of the times it appeared. On the other hand, the three least important roles perceived by the physicians were withholding diagnosis/prognosis, extending patient’s life for as long as possible and discussing treatment costs (Fig. 2).

## Discussion

Results show considerable variation in physician recommendations for EOL care, some of which can be predicted by physician characteristics. If these results extend to real life, it raises significant concerns as to whether patients are receiving the most appropriate treatment at the EOL. Unfortunately, there are no real world studies that we are aware of to gauge the veracity of the results. Results further show that lack of appropriate knowledge/attitude in some aspects of palliative care, reinforcing the need to focus on palliative care education among non-palliative care physicians.

More experienced physicians were more likely to recommend life-extending treatments to patients without cognitive impairment, possibly due to their greater expertise in use of these treatments. However, with more experience, there is also a realistic appreciation of patient’s prognosis and realization of the apparent futility of such treatments, especially among those with cognitive impairment. Thus experienced physicians become less likely to recommend life-extending treatments to patients with cognitive impairments. Alternatively, this relationship could denote a cohort effect, due to younger physicians being trained in a different medical culture as compared to older physicians. However, physicians trained outside Asia were more likely to recommend these treatments to patients with cognitive impairment. The reason for this is not known. Nevertheless, study results suggest that if all decision making were left to physicians, there would likely be large variation in treatment recommendations even for similarly situated patients. By eliciting and respecting patient preferences, it may be possible to minimize variations in EOL treatments as a result of differences in physician practice styles or their personal characteristics. Formulation of standards or guidelines for care and their promotion by the government or third party payers, perhaps tied to pricing and reimbursement, may also help to reduce practice variation, as would greater levels of utilization review.

As with our findings, previous studies have also found that patients with dementia are less likely to be treated for cancer [20–23]. With an increase in prevalence of dementia, physicians can expect to face these decisions more frequently. It is unclear whether life-extending treatments offer any survival benefit to patients with moderate to severe cognitive impairment, [23] and thus it is difficult to define what course of treatment will achieve the best possible medical outcomes for these patients. This is further complicated by the challenge of eliciting patient preferences, as most patients with serious cognitive impairment lack capacity for decision making and even minor diagnostic and treatment procedures may cause significant distress to the patient. Future studies on this topic and development of guidelines for treatment of ASI among patients with cognitive impairment may help physicians provide clearer guidance to caregivers and work to reduce variation in physician recommendations.

Despite some evidence that age per se does not influence the efficacy of many life-extending treatments, [24–26] we find that physicians are more reluctant to recommend these treatments to older patients as compared to younger patients, given the same prognosis and treatment costs. This is consistent with previous literature reporting an age bias in physician recommendation of cancer treatments and life-sustaining treatments [27, 28]. It may be that older patients are at a greater risk of adverse drug reactions, [29] that physicians believe that older patients may not want as much life-extending treatment because of fatalist attitudes, or because physicians themselves attach lower value to extending life in an older patient compared to a younger patient. Therefore, efforts should be made to encourage physicians to discuss patient’s treatment preferences without being biased by their age.

Treatment costs also influence physician treatment recommendations; however, physicians did not seem to view discussing treatment costs with EOL patients as an important role, possibly because they feel that they do not have the skills, knowledge, or time to do so or because they do not see it as their role.

We further show that despite the presence of well-established guidelines regarding treatment of severe pain, only 50 % of the physicians surveyed responded correctly to a statement regarding appropriate opioid prescription. Furthermore, Singapore trained physicians scored lower compared to physicians trained in many other developed countries. Insufficient exposure to palliative care in the undergraduate medical curriculum in Singapore may have contributed to this. Medical students in Singapore receive only 4 days of training in palliative care in their entire undergraduate medical curriculum, as compared to an average of 14 days in United States [10, 30, 31]. Basic palliative care education and training for medical students, residents, and practicing physicians is needed to fill this lacuna, and one needs to look into the best models for incorporating palliative care in training curricula at various levels.

The study has several limitations. First, it is hypothetical and results may not generalize to real patients. There is also a lack of clinical detail upon which to make decisions; more detail may have generated greater consistency. Moreover, despite promising confidentiality, it is possible that physician responses may have been influenced if they felt that they were being evaluated. It is also possible that framing of some of the statements in the best-worst tasks could have influenced physician responses. For instance, the statement ‘extending patient’s life for as long as possible’ is worded quite strongly and as a result could have been consistently underrated by physicians. Further, despite every effort to expand this survey to all major departments in major public hospitals, some of the departments did not allow or did not have time for the study team to present in their department meetings. However, we have no reason to believe these departments would respond differently. Lastly, due to the absence of a sampling frame of physicians, we invited physicians to participate in the survey by advertising in department meetings and through department heads and colleagues. Due to different modalities of invitation and advertisement, it was not possible to calculate how many physicians received the invitation to do the survey and hence the response rate.

## Conclusion

Results from this study suggest that variation in physician treatment recommendations may be partly related to their own characteristics. If confirmed, this might raise concerns regarding the EOL care being provided to patients. Efforts should be made to better understand the variation in treatment recommendations for similarly situated patients in specific clinical settings and to provide physicians with additional training in key aspects of palliative care management.

## Additional file

Additional file 1:
**Health care system in Singapore.** (DOCX 26 kb)

## Abbreviations

EOL: End-of-life
ASI: Advanced serious illness
