# Supplementary material for: Variation in physician recommendations, knowledge and perceived roles regarding provision of end-of-life care
Source: BMC Palliat Care. 2015 Oct 26;14:52. doi: 10.1186/s12904-015-0050-y (PMC4623295; doi:10.1186/s12904-015-0050-y)
Supplement: Additional file 1: — Health care system in Singapore. (DOCX 26 kb) [file 12904_2015_50_MOESM1_ESM.docx]

**Appendix 1: Health care system in Singapore**

Singapore is a rapidly aging country in South-east Asia. It is well known for its efficient healthcare system.[[1](#_ENREF_1)] A World Health Organization report on comparative health systems in 2000 ranked Singapore sixth globally in terms of overall health system performance in healthcare outcomes well ahead of many developed countries, including the United States. The results are all the more significant as Singapore spends less on healthcare than any other high-income country.[[2](#_ENREF_2)]

**Organization of health care services**: Singapore has a mixed delivery model. Eighty percent of primary healthcare services are offered by private medical clinics; whereas the remaining is delivered by government polyclinics. In contrast to primary healthcare, public hospitals provide 80% of hospital care.[[3](#_ENREF_3)] Hospital care consists of inpatient, outpatient and emergency services. The public hospitals have substantial autonomy over management and financial decisions.[[4](#_ENREF_4)] The hospitals compete with each other for patients and hospital bill sizes are published by the ministry to highlight the variation in costs among the hospitals, and to keep up the competition to keep prices low. Finally, step-down care (e.g. nursing homes, community hospitals and hospices) is provided mainly by voluntary welfare organizations, most of which are heavily funded by government. [[5](#_ENREF_5)]

**Financing of health care:** While the Singapore government subsidizes up to 80% of the total bill in acute public hospital wards to keep basic healthcare affordable, at the same time, it also emphasizes individual responsibility towards health.[[6](#_ENREF_6)] This is achieved through the “3M” (Medisave, Medishield and Medifund) system. Medisave is a medical saving account that can be used to pay part of hospital bills and outpatient treatments for members and their immediate family members. In addition, Medisave can be used to pay premiums for Medishield, a high deductible catastrophic insurance plan. MediShield also enables members to settle part of the expenses arising from prolonged hospitalization and certain outpatient treatments for serious illnesses incurred in medical institutions involved.[[7](#_ENREF_7), [8](#_ENREF_8)] Many middle and high income residents have also supplemented their basic coverage through Medishield with integrated private insurance policies for treatment in private sector. In 2015, Medishield is being replaced by Medishield Life that will provide healthcare coverage for the very old (in contrast to Medishield that had an age cap of 85 years) and those with pre-existing illnesses. [[9](#_ENREF_9)]

Finally, Medifund is an endowment fund set up by Singapore government to help residents whose Medisave and MediShield are inadequate to pay the medical expenses. It is not an entitlement program; payments are distributed on a case-by-case basis. The application for Medifund is subject to the approval. [[8](#_ENREF_8)]

Medisave currently accounts for 8% of national health care expenditure while Medishield and Medifund together account for not more than 2%. The rest comprise employer benefits (35%), government subsidies (25%), out-of-pocket payment (25%), and private insurance (5%). [[10](#_ENREF_10)]

**References**:

1. Ham C. Learning from the tigers: stakeholder health care. The Lancet. 1996;347(9006):951-3. doi:<http://dx.doi.org/10.1016/S0140-6736(96)91421-X>.

2. World Health Organization. Health Systems: Improving Performance. Switzerland2000.

3. Bai Yu SC, Li Xiaofeng, and Liu Feifei. Healthcare System in Singapore.

4. Lim MK. Health care systems in transition. II. Singapore, Part I. An overview of health care systems in Singapore. Journal of public health medicine. 1998;20(1):16-22.

5. Haseltine WA. Affordable Excellence: The Singapore Healthcare Story. Ridge Books and Brookings Institution Press; 2013.

6. Barr MD. Medical savings accounts in Singapore: a critical inquiry. Journal of health politics, policy and law. 2001;26(4):709-26.

7. Central Provident Fund Board. Understanding Medisave and Medishield. Central Provident Fund Board, Singapore. 2013. <http://mycpf.cpf.gov.sg/CPF/my-cpf/Healthcare/PvdHC3.htm>. Accessed April 29 2013.

8. Ministry of Health. Schemes & Subsidies. Singapore. 2015. https://[www.moh.gov.sg/content/moh_web/home/costs_and_financing/schemes_subsidies.html](http://www.moh.gov.sg/content/moh_web/home/costs_and_financing/schemes_subsidies.html). Accessed September 1 2015.

9. Health Mo. Medishield Life. Singapore. 2013. <http://www.moh.gov.sg/content/moh_web/medishield-life.html>.

10. Lim MK. Shifting the burden of health care finance: a case study of public-private partnership in Singapore. Health Policy. 2004;69(1):83-92.
